# High-Value Care Inclusion in the Preclinical and Clinical Years of Medical School: A Narrative Review and Descriptive Experience at a Single Institution

**DOI:** 10.7759/cureus.103932

**Published:** 2026-02-19

**Authors:** Lauren K Storm, John D Salvemini, Micaela L Shields, Sarah K Harris, Jeffrey A Roux, Camille C Couey, Johnny Yang, Caroline G Doherty, John S Overton, Joshua B Jeter

**Affiliations:** 1 Internal Medicine, University of Mississippi Medical Center, Jackson, USA

**Keywords:** choosing wisely, cost-effective, high-value care, hvc, preclinical curricula, value-based care

## Abstract

High-value care (HVC) describes patient-centered management by reducing unnecessary practices and decreasing the overutilization of healthcare resources. Cost-effectiveness is a positive byproduct of its implementation. This principle has recently emerged as a key concept in healthcare, with efforts from several institutions being taken to implement HVC into medical school and residency education. HVC education, however, remains limited in medical school curricula, particularly during the preclinical (first and second) years. As a result, students may be less prepared to apply concepts of value-based care (VBC) during clinical rotations and beyond into clinical practice. Although the implementation of HVC curricula for clinical medical students and residents has been well studied, fewer studies address its integration into preclinical education. Our narrative review examines the existing literature on preclinical and clinical HVC education, exploring already-implemented strategies and initiatives. We further describe HVC education initiatives undertaken at our institution, including the implementation of a first-year selective course, as well as other extracurricular opportunities for medical students designed to bridge the gap between evidence-based research and HVC. Early exposure to HVC learning during the preclinical and clinical years of undergraduate medical training has been suggested to increase awareness of value-based diagnostic and treatment practices and encourage a more focused approach to patient care. Programs not limited to, but including the Mayo Clinic’s Science of Health Care Delivery curriculum and Thomas Jefferson University’s pilot incorporating Choosing Wisely principles, have shown positive outcomes in fostering a deeper understanding of VBC. Despite challenges in implementing HVC education within the traditional curricula and ensuring engagement, we posit that early integration of HVC remains essential for preparing future physicians to deliver patient-centered, cost-effective care.

## Introduction and background

High-value care (HVC) is a growing approach in healthcare that prioritizes maximizing the effectiveness and value of care delivered to patients while enhancing the efficiency of healthcare practices. Over the past decade in the United States, the topic of HVC has gained the attention of many within the medical community [1,2]. HVC is medical care founded on the principle of delivering evidence-based, patient-centered treatment that optimizes health outcomes, minimizes harm, and reduces unnecessary costs. By focusing on outcomes such as reducing unnecessary interventions and maintaining cost-effectiveness, HVC remains boundless across specialties and interprofessional disciplines [3]. This model not only seeks to enhance the patient's experience but also promotes sustainability in healthcare systems, seeking long-term benefits for both individuals and the greater healthcare system [4]. In essence, HVC serves as a framework aimed at minimizing redundant practices, thereby improving both time- and cost-efficiency throughout healthcare settings [3]. The concept of value can be measured through a balance of resource utilization, time spent, and financial cost to both the patient and the institution [2]. A central component of HVC explores whether a given diagnostic test or therapeutic intervention provides meaningful clinical benefit that justifies its cost and, more importantly, aligns with the patient’s own goals and care plan [1,2]. The American College of Physicians (ACP) and similar organizations have clearly articulated the principles of HVC and provided a wealth of clinical applications [1,5,6]. Many institutions have already taken strides to incorporate HVC into their clinical curricula. However, the integration of HVC at the preclinical stage has lagged [7,8,9]. This gap in early education may hinder the ability of future healthcare professionals to adopt these principles effectively, potentially delaying their understanding of how to deliver value-driven care from the outset of their training. 

Despite the recognition of HVC by the medical community, the concept is still slowly progressing within medical education and postgraduate training [9,10,11]. Much of the existing research on HVC has focused on its implementation during clinical (commonly, third and fourth) years [7,8,12]. However, there is a noticeable gap in research concerning the early integration of HVC into the preclinical (commonly, first and second) years. Integrating HVC into medical education has been heterogeneous at best, with a large portion of HVC education thus far implemented at the postgraduate level [1]. It is beneficial to implement HVC education within specialty-specific residencies. It is also likely beneficial to implement the HVC curriculum during the preclinical years to establish a foundational understanding of HVC and potentially increase its applicability and efficacy in future practice [7,8,9,12]. Undergraduate medical education is continuously evolving. For instance, preclinical curricula vary by institution across the United States, ranging from traditional subject-based years to system-based approaches to earlier clinical clerkships. Coupled with the growing acceptance of HVC in medicine, we posit that introducing HVC concepts early in medical education would be valuable in shaping students' approaches to medical care [5,8,9,12]. It may help instill the importance of efficiency, resource management, and cost-effectiveness before students encounter real-world clinical scenarios. Through this literature review, we aim to explore and summarize existing research on the impact of HVC education in both clinical and preclinical years, with emphasis on the gaps seen within preclinical and clinical years. We further seek to highlight the approaches taken to better incorporate HVC topics within our own medical school curricula.

## Review

Medline/PubMed and Embase databases were queried for this narrative review. The following keywords were used: “medical education” and “high value care”. Only published studies in English from 2010 to 2024 were included. Both prospective and retrospective studies were considered. Meta-analyses, systematic reviews, and review articles were included, while conference abstracts, letters to the editor, and commentaries were excluded. Our search criteria returned 367 published studies. Research that focused on medical education for preclinical and clinical medical students was included, whereas literature detailing graduate medical education was excluded. Only seven studies examined preclinical medical education, and all of these were included. A total of 91 studies detailing HVC education for clinical medical students were obtained. As this review seeks to focus on the gap in literature detailing preclinical curricula, five selected studies concerning the clinical years of medical school are discussed below. Due to the overwhelming abundance of literature already available for HVC education for the clinical years, we included five studies that provided unique concepts for comparison of curricula in the latter years.

Benefits of HVC in medical education and current outcomes of clinical curriculum implementation

Healthcare is evolving with advancing technology and innovations. Similarly, patient and institutional costs have largely been increasing. Considering that HVC practices remain beneficial in this context, the American Board of Internal Medicine Foundation’s Choosing Wisely campaign seeks to educate the healthcare community to reduce unnecessary tests and services. This is evidently represented by their “Things We Do for No Reason” catalog, which examines common clinical scenarios in which healthcare services are commonly ordered that do not change medical management and may drive up healthcare costs [3]. For example, the common practice of prescribing docusate for constipation is supported by weak evidence without significant benefit over placebo [13]. Providing only necessary care - while minimizing harm to the patient and aligning management with the patient’s own goals and wishes - lies at the forefront of the HVC movement. This could involve stopping unnecessary vital sign checking overnight in hemodynamically stable patients or limiting the use of serial ammonia levels for patients with hepatic encephalopathy, suggesting that these values do not guide clinical management [14]. If continuous monitoring is unlikely to change outcomes, it is important to question what benefits these two forms of management hold for the patient’s overall care.

Physicians must further account for the growing tab that will later be the patient’s responsibility. HVC practices aim to provide tailored care through resource stewardship, and these decisions largely fall on the provider. Value has been previously defined by Porter et al. as health outcomes achieved per dollar spent [2]. As such, HVC has gained more attention due to its potential to provide cost-effective care that is not medically inappropriate or insufficient. In the United States, the cost of healthcare spending as a percentage of gross domestic product has more than doubled, while per capita healthcare spending has increased nearly fivefold [1]. Expenditure on healthcare is imposing an increasing burden on the budgets of both federal and state governments and patients without producing commensurate improvements in health or quality of care [4]. Medical literature shows that instilling awareness of the high costs associated with various imaging, bloodwork, medications, and beyond is more compelling for early medical education [15]. By implementing the cost-focused aspect of medicine early on, students would be better equipped to comprehend the costs associated with testing and the inherent characteristics of the proposed medical condition [1]. Adopting a cost-conscious approach to diagnostics can train the next generation of physicians to not only rely on clinically acquired heuristics but also develop a critical mindset and a refined differential diagnosis. By maintaining a more focused differential diagnosis, we can avoid unnecessary reliance on imaging or laboratory tests ordered based on uncertainty, thereby reducing certain healthcare costs for patients. Focusing the diagnostic workup on studies that are targeted to provide clinically actionable change may further minimize healthcare costs for patients and facilities. Although HVC prioritizes the reduction of unnecessary healthcare practices, we also advocate for strategies that may initially be more expensive but yield long-term cost savings and, more importantly, deliver substantial clinical benefits. Examples include the use of continuous glucose monitors (CGMs) versus self-monitoring of blood glucose (SMBG) and the adoption of large panel next-generation sequencing (LP-NGS) compared to single-gene testing (SGT). Despite higher upfront costs of CGMs, their efficiency can reduce hospitalizations due to the risk of severe hypoglycemia episodes in type 1 diabetics [16]. A study focused on gene sequencing looked at the cost-effectiveness of LP-NGS compared to SGT for patients with advanced non-small cell lung cancer (aNSCLC) and suggested that the use of LP-NGS to guide first-line treatment decisions is clinically more appropriate (more likely to identify alterations and subsequently allocate patients to clinically appropriate therapy) and provides a dominant cost-effectiveness treatment strategy over five years for patients with newly diagnosed aNSCLC in the United States [17]. These studies indicate that, despite higher initial costs, both approaches can improve patient outcomes and ultimately reduce long-term healthcare costs.

As HVC and quality improvement initiatives gain traction, the challenge remains: how to implement an effective and innovative curriculum nationwide? In comparison to the minuscule literature available on HVC curriculum in preclinical years, tangible efforts have been made by several institutions to integrate HVC into clinical undergraduate education. A common tactic appears to be the implementation of HVC curricula into the third-year internal medicine (IM) clerkship. The Johns Hopkins University School of Medicine implemented a blinded intervention and control group during the third-year IM clerkship to assess how the HVC curriculum affects medical students' ordering practices and standardized patient (SP) exams, with the end goal of increasing medical students' perception of HVC education and reducing patient care spending. At the end of the nine-week clerkship, the intervention group had significantly lower Medicare-allowable fees and higher confidence in the quality of their HVC education than the non-intervention group [18]. A different approach was noted at the University of Illinois College of Medicine in Peoria, where HVC didactics were combined with virtual standardized patient (VSP) and SP cases based on the American College of Physicians (ACP) HVC curriculum [19]. This randomized control trial showed an increase in cost-conscious care regardless of the simulation modality (VSP vs. SP). A qualitative example of HVC integration into the IM clerkship was seen at the University of New Mexico School of Medicine, where fourth-year medical students on a four-week IM sub-internship were given four one-hour sessions that applied Choosing Wisely principles to clinical examples and were surveyed three times: before the curriculum, after the curriculum, and just before graduation, with results showing an increase in positive attitudes, perception of practicing HVC, and confidence in forming value-based recommendations [20].

Baylor College of Medicine and the Vanderbilt School of Medicine are examples of institutions that took broader approaches that go beyond the constraints of the IM clerkship. At Baylor, online modules designed to improve evidence-based test ordering were implemented in five core clerkships: family medicine, emergency medicine, IM, neurology, and obstetrics and gynecology. Across all five core clerkships, post-test scores on laboratory stewardship were higher than pre-test scores [21]. In comparison to Baylor’s virtual approach, Vanderbilt took a more intensive approach with their four-week post-clerkship elective titled “High Value Care: In Policy and Practice.” Based on Porter’s six major elements of a value-based system, the course was an amalgamation of lectures, online modules, a weekly book club, and quantitative and qualitative metrics, including quizzes, a final exam, a final presentation, a preceptor assessment, and student participation [22]. While these studies differed in modality and measured outcomes, they all supported the larger consensus that exposure to HVC principles improves awareness, perception, and practice of value-based, patient-centered care. 

Despite positive outcomes seen with HVC implementation in clinical curricula, the progress in preclinical medical curricula is less substantial. Bartlett and Huerta of the University of New Mexico School of Medicine, however, implemented an innovative experiment that reached both preclinical and clinical groups. Bartlett and Huerta published results detailing their experiential extracurricular group that was created with the purpose of quality improvement and patient safety. Their findings showed a statistically significant improvement in the ability of 22 first through fourth year medical students to incorporate the concepts taught during the series into their future practice [23]. While this study remained limited to the extracurricular setting and sample size, Bartlett and Huerta recognized the need for curricular application to better integrate the relationship between HVC, clinical skills, and basic science [23]. By instilling HVC principles in preclinical students, third-year students can enter the clinical landscape with an adjusted perspective. Encouraging broad differentials fosters critical thinking, but this should be grounded in evidence-based practice. Although rare diagnoses - so-called “zebras” - are intriguing, the shift from preclinical to clinical training requires a focus on the essential question: “What do we need to know that will affect management?” rather than broadly “What tests can we order?” [5]. By teaching these concepts early, medical students may be more likely to approach clinical settings with a mindset that blends critical thinking with an awareness of the personal and cost implications for patients.

Current outcomes of preclinical curriculum implementation 

The integration of value-based care (VBC) into the preclinical years of medical school education is increasingly recognized as a vital component in the initiative toward empowering future physicians to practice a culture of patient-centered, cost-effective care. Currently, there is no standardized approach to the incorporation of HVC into the preclinical curriculum, and the available outcome data are sparse. However, several institutions have taken a variety of different routes with overall positive outcomes that demonstrate utility for HVC education at the preclinical level. 

Two valuable studies that focus on HVC curriculum implementation arise from Mayo Clinic School of Medicine’s Science of Health Care Delivery (SCHD) curriculum and from Penn State College of Medicine. Mayo Clinic School of Medicine (MCSM) and Arizona State University (ASU) created a partnership to develop the SCHD curriculum due to a mutual need between the two institutions: ASU needed a clinical context for its healthcare programs despite not having a medical center; MCSM needed additional knowledge of healthcare delivery in content areas such as population health, healthcare economics, and leadership [10]. A four-year curriculum was designed to align with the “Triple Aim” of healthcare: improving outcomes, enhancing patient experience, and reducing costs [17]. The curriculum covered six domains (person-centered care, population-centered care, team-based care, HVC, healthcare policy, economics, technology, and leadership) and employed a hybrid learning approach that included online modules, classroom discussion, simulations, and experiential learning, providing a robust framework for medical students to understand and apply HVC principles in real-world settings [10]. Positive outcomes were observed with the Barr-Kirkpatrick model, indicating positive learning and behavioral changes associated with HVC concepts [11]. Challenges noted, however, were misalignment of the SHCD curriculum with traditional medical education expectations and lack of faculty buy-in. Since HVC is one aspect of the larger SHCD curriculum, subgroup analysis of specific HVC metrics should be examined to further pinpoint its outcome effects. Building on this concept, successful longitudinal integration was also observed at Penn State College of Medicine, where its Health Systems Science Curriculum, implemented in 2014, demonstrated positive outcomes in incorporating HVC principles. Among the many content domains of this curriculum, the “Science of Health Systems” course provides 105 hours between the first and second years of undergraduate medical education. Since the curriculum’s integration in 2014, over 1,000 Penn State Health patients have been positively impacted by student work [11]. Initial performance gap challenges were met due to educator and student attitudes toward acclimation, tracked using evaluation metrics [11]. Despite the initial roadblocks this curriculum faced, it can serve as a model for how to implement, acclimate, and adjust in the setting of an innovative academic environment. Like the work from Mayo Clinic, this curriculum implementation expands beyond HVC. We recommend future subgroup analysis to pinpoint specific ways to integrate HVC into preclinical curricula. 

Another, albeit less intensive, example was seen at Thomas Jefferson University, where a pilot study was performed that incorporated learning objectives based on Choosing Wisely principles into the undergraduate medical education curriculum. This study sought to understand the most effective strategy to incorporate VBC principles in medical education. Over four years, a total of 700 survey responses were obtained, and formal exam questions based on VBC were incorporated into the first- and second-year exams [6]. The efforts made by this institution yielded positive outcomes, including increased awareness of the Choosing Wisely campaign, enhanced knowledge of VBC principles, and improved performance on formal VBC exam questions. These positive findings demonstrate that the Choosing Wisely material may successfully instill early motivation in students to remain cognizant of the value of healthcare in future practice. 

A focused student-led approach was implemented at the University of Vermont’s Larner College of Medicine. Due to the lack of a standardized approach and challenges with curriculum overhauls, students involved in the Students and Trainees Advocating for Resource Stewardship (STARS) Program worked with faculty to integrate Choosing Wisely recommendations into preclinical pathophysiology course topics. Positive outcomes were observed in survey assessments, which showed an increase in the number of students who reported an increased awareness of HVC and an increased likelihood of applying HVC concepts in their future medical practice [7]. This practical approach highlights the potential of sustainable, student-led approaches to HVC models. A challenge with this approach, however, remains a dependency on faculty involvement and student engagement. However, this model may offer a more feasible and effective approach to HVC education in the absence of an extensive formal curriculum change or implementation.

While HVC seeks to reduce unnecessary practices, cost reduction is an added benefit. At the Zucker School of Medicine at Hofstra/Northwell, problem-based learning (PBL) courses regarding healthcare cost topics were integrated into the curriculum for first- and second-year medical students [8]. Students were prompted to discuss cost considerations in clinical practice during these PBL courses for grading purposes. PBL faculty facilitators noted that cost conversations were increasingly initiated by students as they transitioned to their second year. This study did not concentrate on HVC topics but rather the secondary financial arm of HVC. Considering this, Zucker’s curricular addition may increase awareness of cost and, more importantly, provide groundwork for developing clinical practice patterns at an early stage in students' education that consider cost in patient care. 

The Student High Value Care (sHVC) Initiative at the Icahn School of Medicine at Mount Sinai represents a longitudinal model for student-led implementation and scholarship in HVC. It focuses on value-based quality improvement projects in collaboration with hospital faculty, enabling students to actively participate in the development and execution of interventions designed to improve care efficiency and reduce unnecessary costs. This initiative has been successful, with nine of the 10 projects showing statistically significant improvements in their measured outcomes [24]. In addition to the provision of mentorship and scholarship opportunities, the sHVC Initiative provides a practical and effective framework that integrates HVC into preclinical medical school education and showcases HVC’s potential to drive culture change and improve healthcare delivery. 

A Johns Hopkins School of Medicine study examined whether an HVC course could help first-year medical students acquire skills for cost-effective practice, offering a unique approach not previously mentioned in this review. A total of 118 first-year medical students at Johns Hopkins School of Medicine between 2013 and 2017 enrolled in an HVC course that provided the initial framework to practice cost-conscious clinical medicine [12]. The curriculum was evaluated by comparing the performance of students who completed the course with the performance of students without training, using an SP encounter on musculoskeletal back pain and how to approach a patient's request for imaging. Students who enrolled in the HVC course were more likely to assure patients that back pain was a simple strain and were less likely to ask for preceptor help on how to proceed with management (p = 0.007) [12]. No differences regarding initial imaging management were shown for students enrolled in the HVC course who had not received the training yet versus students not enrolled. This study was limited to one clinical situation but is valuable for demonstrating how a case-based HVC course can help first-year students develop skills essential for cost-effective practice, a key pillar of HVC. 

Despite promising outcomes seen thus far in the literature, the implementation of HVC curricula in preclinical education still faces challenges. Faculty plays a large role in the implementation of HVC curriculum changes. Traditional expectations of medical education have a large effect on both student and faculty outcomes [7]. A large barrier in implementing value-based curricula is the mixed receptivity and engagement from students, which manifests as a misconception between expectations of traditional medical school education and the time spent in VBC-centered education [25]. While the implementation of VBC at various institutions over the past decade is encouraging, the shift toward a more holistic approach to academic medicine proves difficult due to a larger cultural challenge that requires time, continuous modifications, and a cultural transformation of our educational system [11]. Cultural barriers to educational transformation may also occur. Challenges, including a lack of faculty development and availability [9] and difficulty in assessing long-term outcomes, may further hinder large-scale implementations across institutions. These obstacles may delay or limit the widespread adoption of innovative curricula; however, the existing literature - though limited - demonstrates that institutions are actively exploring effective approaches to HVC education. Table 1 provides a concise overview of the strategies described above.

**Table 1:** Overview of current outcomes of preclinical curriculum implementation HVC: High-Value Care; SCHD: Science of Health Care Delivery; STARS: Students and Trainees Advocating for Resource Stewardship; PBL: Problem-Based Learning; sHVC: Student High Value Care; VBC: Value-Based Care; MS1: First-Year Medical Student; MS2: Second-Year Medical Student

Institution	Curriculum / Intervention	Educational Approach	Key Outcomes	Challenges / Limitations	Reference
Mayo Clinic School of Medicine	SCHD Curriculum	Four-year, longitudinal curriculum aligned with the "Triple Aim"; hybrid learning including online modules, classroom discussion, simulation, and experiential learning; HVC as one of multiple domains	Positive learning and behavioral outcomes per the Barr-Kirkpatrick model; improved understanding of HVC principles	Misalignment with traditional medical education expectations; lack of faculty buy-in; HVC represents only one component - limited HVC-specific outcome analysis	[10]
Penn State College of Medicine	Health Systems Science Curriculum (2014)	Longitudinal integration; 105 hours of instruction between MS1-MS2; includes HVC among multiple domains	Over 1,000 patients positively impacted by student work; improved acclimation over time	Initial performance gaps due to student and educator attitudes; curriculum extends beyond HVC, limiting subgroup analysis	[11]
Thomas Jefferson University	Choosing Wisely-Based Pilot Curriculum	Integration of VBC learning objectives and exam questions into preclinical years	Increased awareness of Choosing Wisely; improved VBC knowledge and exam performance	Less intensive intervention; primarily knowledge-based outcomes	[6]
University of Vermont (Larner College of Medicine)	STARS Program	Student-led integration of Choosing Wisely recommendations into preclinical pathophysiology courses	Increased student awareness of HVC; greater intent to apply HVC principles in future practice	Reliance on student engagement and faculty participation; sustainability dependent on continued involvement	[7]
Zucker School of Medicine at Hofstra/Northwell	Cost-Focused PBL	PBL cases incorporating healthcare cost considerations into grading	Increased initiation of cost-related discussions by students, especially in the second year	Focused on cost awareness rather than a comprehensive HVC framework	[8]
Icahn School of Medicine at Mount Sinai	sHVC Initiative	Longitudinal, student-led quality improvement projects with faculty mentorship	9 of 10 projects demonstrated statistically significant outcome improvements - strong experiential learning	Requires sustained mentorship and institutional support	[24]
Johns Hopkins School of Medicine	First-Year HVC Course	Case-based curriculum with standardized patient encounters	Improved student confidence and communication in cost-conscious care (e.g., imaging requests)	Limited to a single clinical scenario; no difference in imaging decisions	[12]

Our institutional experience in implementing HVC in the curriculum 

Both students and faculty have made positive strides in integrating HVC into our medical school curriculum. This effort began in early 2021, thanks to the leadership of an IM physician and HVC advocate. At that time, our institution provided a traditional medical school curriculum (i.e., the basic sciences in the first two years, followed by two clinical years). An optional HVC selective was created for first-year medical students as part of the one non-basic science course, Introduction to the Medical Profession, in which 10 students enrolled. This selective laid the foundation for incorporating HVC principles early in medical education, helping preclinical students grasp the importance of VBC from the outset of their training. This course combines lectures with group presentations, in which students propose HVC initiatives tailored to our institution and community. It offers a hands-on experience that not only educates students about VBC but also encourages them to think critically about applying these practices in real-world settings. To assess the course's effectiveness, a pilot pre- and post-course survey was administered covering foundational HVC topics. Post-course results demonstrated overall improvement in students' awareness of HVC concepts compared to the pre-test. Since then, the HVC selective has continued to expand and evolve, even as our curriculum transitioned to system-based in the fall of 2023. Future studies at our institution aim to expand upon the preliminary survey provided to the first cohort of students to more precisely define tangible metrics regarding awareness, understanding, and application of HVC principles in clinical practice. 

Beyond the classroom, our institution offers extracurricular opportunities that reinforce the principles of HVC. The inclusion of our students in the nationwide STARS program and the activity of the HVC Interest Group are two important components. Our physician mentor played a pivotal role in not only ensuring that HVC principles were integrated as a component of our preclinical medical education but also our institution’s inclusion in the STARS program and the establishment and success of the HVC Interest Group. His leadership and dedication have been instrumental in advancing the curriculum and fostering a deeper understanding of VBC among students. The STARS program, a derivative of the Choosing Wisely campaign, has been an invaluable resource for medical students, helping them engage with key concepts of resource stewardship and VBC. Each academic year, the existing committee selects four new medical students to join the committee through an application process that opens each fall semester. Since the beginning of the STARS initiative at our university, application submissions have continued to increase, which exemplifies the growing interest of HVC among medical students at our university. Each year, the four new STARS members work directly with past cohorts. All can participate in collaborative meetings, leadership summits, and the development and presentation of research deliverables. These experiences deepen the understanding of HVC and help students develop leadership skills needed as future physicians. As part of the STARS program, medical students can collaborate with peers nationwide, driving national initiatives that enhance medical education with the goal of providing high-quality, effective patient care. 

The HVC Interest Group at our institution serves as another important platform for first through fourth year medical students to learn about and actively participate in HVC activities. The HVC Interest Group was created by the STARS cohort during the fall of 2023. The group was established to create a supportive and collaborative environment where students can explore HVC topics and events in a cohesive and comfortable setting. Membership grew to over 50 medical students, mostly preclinical, within six months. This platform provides an opportunity for students not involved in the STARS program to develop and implement their ideas for integrating HVC into their medical school experience. The HVC Interest Group meets twice per semester and features various activities, including lectures by clinicians from different specialties who promote HVC, discussions on innovative techniques to implement HVC into clinical practice, and dedicated sessions for HVC educational learning. Collectively, these two extracurricular initiatives have been successfully integrated into the extracurricular realm of our medical education, with a dual focus on education and practical application, thereby increasing student awareness and engagement with the HVC initiative. 

Most recently, our institution introduced the first annual HVC Research Event to link HVC principles with evidence-based medicine. The STARS cohort of 2026 strategically organized this research poster event in the fall of 2024. The primary goal was for students to demonstrate how HVC can be effectively integrated into clinical practice through their research. The initial feasible goal was to provide a platform for 10 poster presentations. Impressively, 32 research projects were submitted and presented, indicating that while research deliverables are important for medical students, the application of research can frequently be applied to HVC approaches for clinical practice. Retrospective chart review studies, case reports, quality improvement projects, and other modalities were presented. Non-presenting classmates and faculty fostered an engaging environment for discussion and learning. This event highlights the meaningful progress our university has made in creating an environment that educates medical students on the importance of HVC. The authors hope this encourages other institutions regionally and nationally to take similar measures. This may also reflect the increasing interest in HVC applications in medicine, particularly when combined with research.

## Conclusions

HVC is emerging as a useful initiative nationwide, impacting medical students, residents, and clinicians alike, with hopes of becoming an integral way healthcare is taught and practiced. This literature review examines the current research in HVC education during the preclinical years of medical school, investigates how institutions have effectively integrated HVC into their curricula with practical initiatives, and provides an in-depth perspective of our institution's efforts to advance HVC principles. For HVC to thrive as a nationwide initiative in medical education and beyond, medical students, residents, and faculty must embrace a shared commitment to change and innovation in healthcare delivery. Most literature on HVC curricula integration is isolated to the clinical years, with scant literature available on implementation into the preclinical years. The authors recognize the gap in preclinical HVC education and promote its utility at an early stage of medical education. 

This review is a vessel for us to emphasize that achieving a shift toward prioritization and practice of HVC necessitates the collective engagement and support of all medical students, mentors, and faculty alike. Our university is still working toward fully integrating HVC practices into both the preclinical curriculum and clinical years of medical school, with significant room for continued improvement. Like the other institutions highlighted throughout this paper, we aim to inspire action toward meaningful change. We support prospective studies to determine the impact of early HVC education in medicine and to provide valuable insights into effective models for its implementation within the preclinical years of medical education.
